# Cardiovascular disease in relation to diabetes status in immigrants from the Middle East compared to native Swedes: a cross-sectional study

**DOI:** 10.1186/1471-2458-13-1133

**Published:** 2013-12-05

**Authors:** Louise Bennet, Carl-David Agardh, Ulf Lindblad

**Affiliations:** 1 Department of Clinical Sciences, Lund University, Malmö, Sweden; 2 Center for Primary Health Care Research, Region Skåne and Lund University, Malmö, Sweden; 3 Unit of Vascular Diabetic Complications, Lund University, Skåne University Hospital, Malmö, Sweden; 4 Department of Primary Health Care, Institute of Medicine, The Sahlgrenska Academy, University of Gothenburg, Gothenburg, Sweden; 5 Center for Primary Health Care Research, Clinical Research Centre, Building 60, Floor 12, Skåne University Hospital, Jan Waldenströms gata 37, Malmö 205 02, Sweden

**Keywords:** Type 2 diabetes, Cardiovascular disease, Migration, Middle East

## Abstract

**Background:**

Type 2 diabetes is highly prevalent in immigrants to Sweden from Iraq, but the prevalence of cardiovascular disease (CVD) and its risk factors are not known. In this survey we aimed to compare the prevalence of CVD and CVD-associated risk factors between a population born in Iraq and individuals born in Sweden.

**Methods:**

This population-based, cross-sectional study comprised 1,365 Iraqi immigrants and 739 Swedes (age 30-75 years) residing in the same socioeconomic area in Malmö, Sweden. Blood tests were performed and socio-demography and lifestyles were characterized. To investigate potential differences in CVD, odds ratios (ORs) with 95% confidence intervals (CIs) were estimated by multivariate logistic regression analysis with adjustment for metabolic, lifestyle and psychosocial risk factors for CVD. Outcome measures were odds of CVD.

**Results:**

There were no differences in self-reported prevalence of CVD between Iraqi- and Swedish-born individuals (4.0 vs. 5.5%, OR 0.9, 95% CI 0.4-1.8). However, the prevalence of type 2 diabetes was higher in Iraqi compared to Swedish participants (8.4 vs. 3.3%, OR = 4.2, 95% CI 2.6-6.7). Moreover, among individuals with type 2 diabetes, Iraqis had a higher prevalence of CVD (22.8 vs. 8.0%, OR = 4.2, 95% CI 0.9-20.0), after adjustment for age and sex. By contrast, among those without diabetes, immigrants from Iraq had a lower prevalence of CVD than Swedes (2.2 vs. 5.5%, OR = 0.6, 95% CI 0.3-0.9).

Type 2 diabetes was an independent risk factor for CVD in Iraqis only (OR = 6.8, 95% CI 2.8-16.2). This was confirmed by an interaction between country of birth and diabetes (*p* = 0.010). In addition, in Iraqis, type 2 diabetes contributed to CVD risk to a higher extent than history of hypertension (standardized OR 1.5 vs. 1.4).

**Conclusions:**

This survey indicates that the odds of CVD in immigrants from Iraq are highly dependent on the presence or absence of type 2 diabetes and that type 2 diabetes contributes with higher odds of CVD in Iraqi immigrants compared to native Swedes. Our study suggests that CVD prevention in immigrants from the Middle East would benefit from prevention of type 2 diabetes.

## Background

Cardiovascular disease (CVD) is the most common cause of death in Sweden [1]. One of the strongest risk factors for CVD is type 2 diabetes (T2D) and they both share causal pathways through lifestyle, metabolic and socioeconomic risk factors identified in the INTERHEART study [2]. T2D is increasing worldwide for many reasons, such as older age and environmental factors (e.g. increasing body weight due to physical inactivity and calorie-dense food), but genetic, psychosocial and socioeconomic risk factors also contribute to the increased risk [3-5]. Migration and urbanization have been shown to increase the prevalence of risk factors for CVD [6,7] and to increase the diabetes prevalence in immigrants from the Middle East [8], but the underlying mechanisms are largely unknown.

In Sweden, the second largest group of immigrants comprises those born in Iraq, and in Malmö, the third largest city in Sweden, Iraqis represent the largest immigrant group [9]. Since immigrants to Sweden from the Middle East have been identified as a group at high risk of diabetes [8], and since studies of diabetic complications such as CVD in this group are scarce, the aim of this survey was to study CVD prevalence and CVD risk factor profiles in a population of first-generation immigrants born in Iraq and to compare them with individuals born in Sweden.

## Methods

### Study population

#### Participants and methods

**Figure 1:**
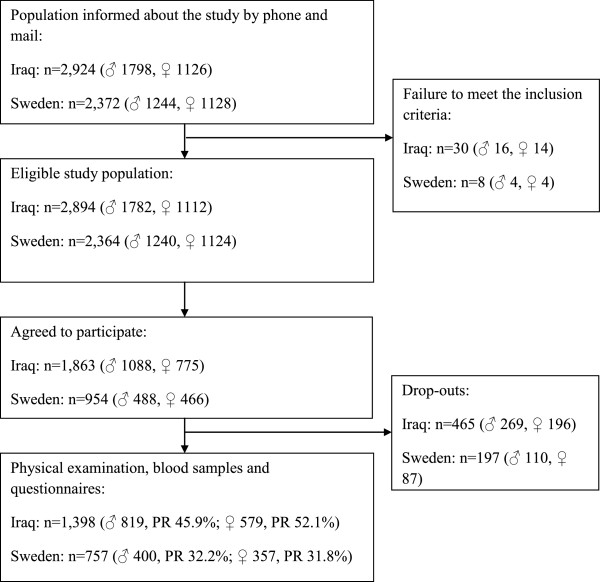
**Flow chart of the recruitment of study participants by country of birth.** PR, participation rate.

Malmö is a multicultural city in southern Sweden, 32% of whose residents in 2011 were born abroad [9]. The largest immigrant group in Malmö consists of people born in Iraq, who collectively account for almost 9,000 of Malmö’s ~300,000 inhabitants. Residents of Malmö born in Iraq or Sweden who were 30 to 75 years of age were randomly selected from the census register and invited by mail and phone to participate in a population-based survey that included a physical examination, fasting blood tests, an oral glucose tolerance test (OGTT) and assessment of family history of diabetes, present medication, chronic diseases and lifestyle habits using questionnaires. We aimed to achieve similar sex and age distributions in the Swedish-born and Iraqi-born groups. Participants categorized as “born in Sweden” included second-generation immigrants born in Sweden. Participants with severe physical or mental illness did not participate in the study. Examinations were conducted between February 1, 2010 and December 21, 2012. Figure 1 shows a flow chart illustrating the recruitment of participants.

#### Measurement of exposure

*Physical examination* Trained Swedish- and Arabic-speaking research nurses conducted standard physical examinations. Participants were instructed not to consume tobacco or eat or drink anything but water after 10 pm the day before testing and to bring a record of their current medications. *Blood pressure* was measured in the supine position after five minutes’ rest and with the arm at heart level. The mean of two measurements, taken one minute apart, was used in analyses. *Body height* was measured to the nearest centimetre using a wall-mounted stadiometer. *Body weight* was measured to the nearest kilogram with participants wearing light indoor clothes but no shoes using a calibrated electronic scale (Coline, 34-5062 RTC3010, China). *BMI* was calculated as weight (kg) divided by height squared (m^2^). *Waist circumference* was measured to the nearest cm in a standing position after a gentle expiration. A tape measure was placed around the bare midriff of each participant and the waist circumference measured midway between the lower border of the rib cage and the superior border of the iliac crest [10].

*Blood samples* were collected in the morning and analyzed continually during the study. Plasma cholesterol and triglyceride levels were determined using enzymatic methods (Bayer Diagnostics) [11], and plasma high-density lipoprotein (HDL) and low-density lipoprotein (LDL) levels were measured enzymatically using a Cobas-® 6000 analyzer (Mannheim GmbH, Germany). Serum insulin levels were determined using a radioimmunoassay (Access^©^ Ultrasensitive Insulin, Beckman Coulter, USA) [12]. C-peptide levels were measured by a one-step immunometric sandwich method using an electrochemiluminescence immunoassay (ECLI) based on a ruthenium (Ru) derivative (Roche). Plasma glucose was measured immediately after sampling using a photometer (HemoCue AB, Ängelholm, Sweden) [13]. HbA1c was estimated by high-pressure liquid chromatography (HPLC) with a VARIANT™ TURBO Hemoglobin A1c Kit 2.0 (Bio-Rad).

We used the risk factors for CVD reported in the INTERHEART study [2], including T2D, history of hypertension, elevated ApoB/ApoA ratio (replaced by elevated plasma (p)-LDL/p-HDL ratio in the present study), abdominal obesity, physical activity (PA) less than 4 hours per week, intake of fruit and vegetables less than once daily, regular alcohol consumption, tobacco smoking and psychosocial factors (i.e. stress, depression, life events and economic difficulties).

*CVD* was self-reported and included history of angina pectoris, myocardial infarction and/or stroke.

*History of T2D* was confirmed by either treatment with oral hypoglycemic agents and/or insulin or by a fasting plasma glucose level ≥7.0 mmol/L [14].

*History of hypertension* was self-reported [2].

*Elevated p-LDL/p-HDL* ratio was a proxy for elevated ApoB/ApoA ratio [2] and was defined as a value in the highest quintile (>3.67).

*Abdominal obesity* was defined as a waist-hip-ratio ≥0.95 in men and ≥0.90 in women [2].

*Physical activity* was estimated using questions developed by the Swedish National Board of Health and Welfare (NBHW) to estimate time spent physically active [15]. Time spent physically active each week doing non-strenuous PA (e.g., walking, cycling or gardening), or undertaking strenuous PA (e.g., jogging, swimming, basketball or football) was estimated by the participants in terms of minutes. As recommended by NBHW, time spent doing strenuous PA was multiplied by two and then added to time spent doing non-strenuous PA [15]. Total minutes per week were transformed to hours per week and participants were then dichotomized with a PA cut-off set at 4 hours per week.

*Intake of fruit and vegetables* was self-reported. Participants were dichotomized into those with intakes of once or more, or less than once per day [2].

*Alcohol consumers* were those who stated that they drink alcohol [2].

*Tobacco smoking* Participants stating that they had never smoked were considered non-smokers and the others were classified as smokers [2].

*Psychosocial factors* were *depression*, *stress*, *life events* and *economic difficulties*[2]; *Depression* was defined as moderate to severe depression, as indicated by a score of >10 points on the Hospital Anxiety and Depression scale [16]. *Stress* was defined by participants stating they often feel stressed in daily life. *Life events* were defined as participants who suffered more than one of the following life events during their lifetime: divorce, illness, experience of war, unemployment, death or illness in the family and experience of a natural disaster. *Economic difficulties on several occasions:* Difficulties in paying for food, rent or bills on more than one occasion during the last 12 months [2].

FINDRISC scores estimate the risk of developing diabetes within the next 10 years [5]. The scores are based on anthropometrical measures, family history of diabetes, lifestyle factors such as physical activity and intake of fruit and vegetables, history of high blood sugar and medication for hypertension.

### Ethical considerations

All participants provided written informed consent and the Regional Ethical Review Board in Lund approved the study (nos. 2009/36 and 2010/561). This investigation conforms to the principles outlined in the Declaration of Helsinki [17].

### Statistical analysis

Analyses were performed using IBM SPSS 21.0 for Windows XP. Skewed variables were log_10_-transformed before analysis to approximate normal distributions. Least squares means were derived after adjustment for age and sex using linear regression, whereas differences in proportions were adjusted for age and sex using logistic regression. Associations with CVD were estimated using multivariate logistic regression analysis. Data are expressed as odds ratios (ORs) with 95% confidence intervals (95% CIs). All tests were two-sided and a *p*-value of <0.05 was considered statistically significant. Regression coefficients for the independent variables were standardized to a unit variance and the proportionality of risk ratios was estimated using standardized odds ratios (SD ORs). Multicollinearity was tested for but was not considered an issue since all variance inflation factors (VIFs) in the multivariate regression models had values <2.1. Goodness of fit was tested using the Hosmer-Lemeshow test, the result of which (*p* > 0.05) indicated a good fit.

## Results

Initially, 1398 Iraqi and 757 Swedish adults participated but information on CVD was missing for some participants. Ultimately, 1365 Iraqi and 739 Swedes were included in this study.

### Prevalence of CVD and T2D

**Figure 2:**
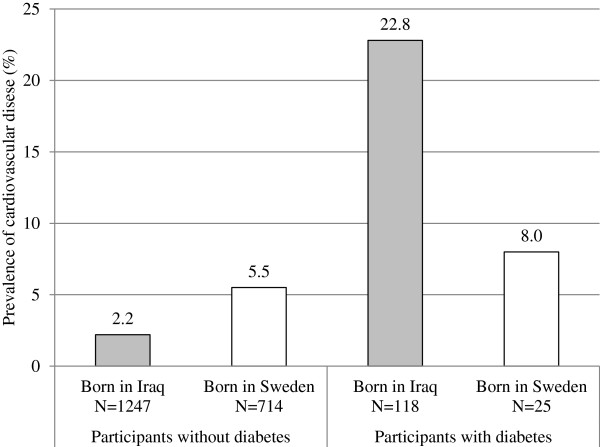
Prevalence of cardiovascular disease in participants with and without diabetes, born in Iraq or Sweden.

There was no difference in the prevalence of CVD (i.e. history of angina, myocardial infarction and/or stroke) in Iraqi versus Swedish participants (4.0 vs. 5.5%, OR 0.9, 95% CI 0.4-1.8). However, in individuals without diabetes (1247 Iraqis and 714 Swedes), the prevalence of CVD was lower in immigrants from Iraq compared to native Swedes (2.2 vs. 5.5%, OR 0.6, 95% CI 0.3-0.9), whereas in participants with diabetes (118 Iraqis and 25 Swedes), the prevalence of CVD was higher in Iraqis (22.8 vs. 8.0%, OR 4.2, 95% CI 0.9-20.0), after adjustment for age and sex (Figure 2).

The prevalence of T2D was higher in Iraqi versus Swedish participants (8.4 vs. 3.3%, OR 4.2, 95% CI 2.6-6.7). Amongst participants without T2D, 38.1% of Iraqis vs. 24.1% of Swedes (*p* < 0.001) had 12 or more FINDRISC points, indicating a moderate (1 out of 6) to high risk (1 out of 3) of developing diabetes within the next ten years [5].

### Basic characteristics of the study participants in relation to CVD status

In participants without CVD, Iraqis had lower blood pressure levels and lower prevalences of alcohol consumption and life events (Table 1). By contrast, they had a more disadvantageous metabolic risk factor profile, with higher plasma levels of fasting glucose, C-peptide and plasma triglycerides, and higher prevalences of T2D, abdominal obesity, physical inactivity and elevated p-LDL/HDL ratio. In addition, psychosocial risk factors such as depression, stress and economic difficulties were more prevalent in Iraqis compared to Swedes among participants without CVD (Table 1).

**Table 1:** Characteristics of the participants with and without cardiovascular disease according to country of birth

	**No cardiovascular disease**			**Cardiovascular disease**		
**Variable**	**Born in Iraq**	**Born in Sweden**	***P***	**Born in Iraq**	**Born in Sweden**	***P***
	***N*** **= 1,311**	***N*** **= 698**		***N*** **= 54**	***N*** **= 41**	
**Age, years**	45.8 (9.4)	49.2 (11.1)	<0.001	57.1 (8.8)	56.3 (10.8)	0.683
**Male sex, *n *** **(%)**	757 (57.7)	370 (53.0)	0.042	44 (81.5)	19 (46.3)	0.001
**Body mass index, kg/m**^**2**^	29.3 (4.5)	27.2 (4.7)	0.001	29.5 (3.9)	27.9 (4.6)	0.129
**Waist-to-hip ratio for men**	1.0 (0.1)	0.9 (0.1)	<0.001	1.0 (0.1)	1.0 (0.1)	0.366
**Waist-to-hip ratio for women**	0.9 (0.2)	0.8 (0.1)	<0.001	0.9 (0.1)	0.8 (0.1)	0.093
**Systolic blood pressure, mmHg**^**a**^	128.6 (16.6)	135.6 (19.5)	<0.001	134.9 (17.7)	137.3 (24.5)	0.249
**Diastolic blood pressure, mmHg**^**a**^	77.8 (10.3)	81.3 (11.3)	<0.001	79.2 (11.3)	80.0 (13.4)	0.182
**Fasting glucose, mmol/L**^**b**^	5.9 (1.4)	5.7 (1.2)	0.054	7.3 (2.7)	5.7 (0.8)	0.839
**C-peptide, nmol/L**	0.8 (0.3)	0.7 (0.3)	<0.001	0.9 (0.4)	0.8 (0.4)	0.583
**Total cholesterol, mmol/L**^**c**^	4.9 (1.0)	5.2 (1.0)	<0.001	4.4 (1.1)	4.9 (1.2)	0.226
**p-low-density lipoprotein, mmol/L**^**c**^	3.2 (0.8)	3.3 (0.9)	0.019	2.7 (1.0)	3.0 (1.0)	0.715
**p-high-density lipoprotein, mmol/L**^**c**^	1.2 (0.3)	1.4 (0.4)	<0.001	1.1 (0.4)	1.5 (0.5)	0.032
**p-triglycerides, mmol/L**^**c**^	1.6 (1.2)	1.3 (0.8)	<0.001	1.9 (1.2)	1.3 (0.6)	0.033
**Education level lower than high school exam, *n *** **(%)**	366 (28.1)	125 (17.9)	<0.001	10 (18.9)	15 (36.6)	0.135
**INTERHEART risk factors **[2]						
**Type 2 diabetes, *n *** **(%)**^**d**^	88 (6.7)	23 (3.3)	<0.001	26 (48.1)	2 (4.9)	0.001
**History of hypertension, *n *** **(%)**^**e**^	146 (11.2)	98 (14.0)	0.465	25 (46.3)	19 (46.3)	0.623
**Elevated p-LDL/p-HDL ratio**^**b**^**, *n *** **(%)**^**f**^	280 (22.7)	102 (15.8)	0.002	9 (18.4)	3 (7.7)	0.385
**Abdominal obesity, *n *** **(%)**^**g**^	783 (60.1)	222 (32.1)	<0.001	36 (67.9)	16 (39.0)	0.006
**Physical activity <4 hours/week, *n *** **(%)**	1004 (84.9)	327 (52.1)	<0.001	35 (85.4)	21 (65.6)	0.058
**Intake of fruit/vegetables < once daily, *n *** **(%)**	676 (52.5)	343 (49.2)	0.703	25 (47.2)	23 (56.1)	0.201
**Alcohol consumption, *n *** **(%)**	229 (18.7)	575 (83.5)	<0.001	17 (40.5)	26 (66.7)	0.006
**Tobacco smoking, *n *** **(%)**^**h**^	310 (23.9)	172 (24.7)	0.307	17 (32.7)	18 (43.9)	0.087
**Depression, *n *** **(%)**^**i**^	194 (16.1)	15 (2.5)	<0.001	5 (11.9)	3 (10.7)	0.981
**Stress, *n *** **(%)**^**k**^	492 (37.8)	138 (19.8)	<0.001	20 (38.5)	16 (39.0)	0.839
**Economic difficulties > once last year, *n *** **(%)**^**l**^	675 (52.2)	104 (14.9)	<0.001	26 (49.1)	10 (24.4)	0.030
**Life events >1, *n *** **(%)**^**m**^	886 (67.6)	605 (86.7)	<0.001	40 (74.1)	36 (87.8)	0.205

Data are presented as means (standard deviation) unless otherwise indicated. Comparisons between means were conducted using linear regression with adjustment for age and sex, while groups were compared using logistic regression with adjustment for age and sex.

^a^Adjusted for age, sex and antihypertensive medication.

^b^Adjusted for age, sex and oral hypoglycemic agents and/or insulin.

^c^Adjusted for age, sex and medication against hypercholesterolemia.

^d^Confirmed by treatment with oral hyperglycemic agents and/or insulin, or by a fasting glucose level of ≥7.0 mmol/L.

^e^Self-reported history of hypertension.

^f^Participants with p-LDL/p-HDL ratios >3.67 (i.e. the highest quintile).

^g^Waist-hip-ratio ≥0.95 in men and ≥0.90 in women.

^h^Previous or current tobacco smokers.

^i^Moderate to severe depression, indicated by >10 points on the Hospital Anxiety and Depression scale [16].

^k^Participants who often feel stressed in daily life.

^l.^Difficulties paying for food, rent and/or bills on several occasions in the last year.

^m^More than one life event during the lifetime.

Among participants with CVD, Iraqi individuals had a 10 times higher prevalence of T2D and, similar to participants without CVD, abdominal obesity and economic difficulties were more prevalent in Iraqis compared to native Swedes (Table 1).

### Odds of CVD

In the total study population, older participants and participants with a history of hypertension or stress had the highest odds of CVD, followed by those with T2D and smokers, after adjustment for the INTERHEART risk factors for CVD [2] (Table 2). We also found interactions between country of birth and T2D (*p* = 0.010) and between country of birth and stress (*p* = 0.037), indicating that the constellation of risk factors for CVD differed between participants born in Iraq and those born in Sweden.

**Table 2:** Risk factors for CVD in accordance with the INTERHEART study

	**Total study population (N = 1,695)**				**Born in Iraq (N = 1,113)**				**Born in Sweden (N = 582)**			
**Risk factor**	**Model I**			**SD OR**	**Model II**			**SD OR**	**Model II**			**SD OR**
	**OR**	**95% CI**			**OR**	**95% CI**			**OR**	**95% CI**		
Born in Iraq	0.7	0.4	1.6		**-**					**-**		
Age	1.1^***^	1.1	1.1	1.8	1.2^***^	1.0	1.1	2.3	1.1	0.9	1.1	
Male sex	1.6	0.8	3.1		2.5	0.8	7.6		1.2	0.4	2.8	
T2D	4.1^***^	2.0	8.3	1.4	6.8^***^	2.8	16.2	1.6	1.2	0.2	6.4	
History of hypertension	3.7^***^	2.0	6.9	1.5	3.3^**^	1.4	7.8	1.4	6.2^***^	2.2	17.3	1.9
Abdominal obesity	0.6	0.3	1.2		0.7	0.2	1.8		0.6	0.2	1.8	
Elevated p-LDL/p-HDL ratio	0.8	0.4	1.8		0.9	0.3	2.5		0.6	0.1	2.7	
Tobacco smoking	2.3^**^	1.3	4.2	1.4	2.2	0.9	5.0		2.0	0.8	5.1	
Alcohol consumption	0.9	0.5	1.8		1.6	0.6	3.8		0.5	0.2	1.3	
Physical activity <4 h/week	1.1	0-6	1.8		1.3	0.4	3.8		1.2	0.5	3.0	
Intake of fruit or vegetables < once daily	1.1	0.6	1.8		0.8	0.3	1.7		1.4	0.6	3.3	
Depression	0.8	0.3	2.0		0.5	0.1	1.7		4.9	0.9	25.4	
Stress	2.5^**^	1.3	4.7	1.5	2.1	0.8	5.1		3.4^**^	1.3	8.8	1.6
Two or more life events	1.2	0.6	2.5		1.7	0.7	4.3		0.7	0.2	2.2	
Economic difficulties	0.7	0.3	1.4		0.4^*^	0.2	0.16	0.6	2.6	0.8	8.9	

^*^p ≤ 0.001 ^**^p ≤ 0.010^***^p ≤ 0.050.

Data was assessed using multivariate logistic regression analysis presenting odds ratios (ORs) and 95% confidence intervals (CIs). Regression coefficients were standardized to a unit variance for the independent variables and the proportionality of risk ratios was estimated using standardized odds ratios (SD ORs).

Immigrants from Iraq with T2D had over six times the odds of CVD as compared to immigrants without T2D (Table 2). In addition, participants with T2D had higher ORs for CVD than those with a history of hypertension (SD OR 1.5 vs. 1.4). Further, economic difficulties reduced the odds of CVD; however, the association was weak and the SD ORs were low. In individuals born in Sweden, participants with hypertension had the highest ORs for CVD, and SD ORs were higher compared to those for Iraqis (1.9 vs. 1.4) (Table 2). Stress also increased the odds of CVD in native Swedes whereas T2D was not an independent risk factor for CVD in this group (Table 2).

### Representativeness of the study sample

The participants in this study were somewhat older compared to the eligible background population (Iraqis by 1.7 years, 95% CI 0.9-2.5, *p* < 0.001; Swedes by 4.5 years, 95% CI 3.5-5.6, *p* < 0.001), but the prevalence of self-reported T2D in participants versus non-participants did not differ significantly (data not shown).

## Discussion

The key finding of this study is that the odds of CVD in immigrants from Iraq are highly dependent on the presence or absence of diabetes. Among individuals diagnosed with T2D, the odds of CVD were more than four times higher in immigrants from Iraq compared to native Swedes, whereas in individuals without diabetes, the odds of CVD in Iraqis were half as high as the odds of CVD in native Swedes. That T2D increased the odds of CVD in Iraqis only was confirmed by an interaction between country of birth and diabetes. Our study suggests that CVD prevention in immigrants from the Middle East in particular would benefit from prevention of T2D.

### CVD in relation to diabetes status and country of birth

Immigrants from Iraq are the second largest immigrant group in Sweden and represent a large proportion of the Swedish population [9]. Cohort studies have reported that the relative risk of coronary heart disease is twice as high in immigrants from Iraq compared to native Swedes [18]. In this study the prevalence of CVD in immigrants from the Middle East was somewhat lower compared to the figure reported from a study conducted in Norway of immigrants from Pakistan (4.0 vs. 7.4%) [19], but the prevalence of CVD in the Swedish-born group corresponds well with the prevalence of CVD in Sweden in general in 2011 (5.5 vs. 5%) [9].

Although T2D is highly prevalent amongst immigrants to Sweden from the Middle East [8] and is a strong risk factor for CVD [20], studies of CVD in relation to diabetes status in non-European immigrants are scarce. In a Norwegian study comparing ethnic Norwegians with an ethnic minority group of immigrants from the Middle East and Asia, in individuals without diabetes, the minority group had an increased risk of CVD as compared to Norwegian subjects, whereas in participants with diabetes, there were no differences in CVD risk between the groups [19]. However, differences in inclusion criteria, settings and study populations make it difficult to draw any conclusions by comparing the studies. Other studies have shown that the incidence of macrovascular complications such as myocardial infarction, angina and stroke in diabetes patients varies not only between different ethnic groups, but also in different settings. In the United Kingdom, South Asians are reported to have a higher prevalence of macrovascular disease compared to White Caucasians [21]. By contrast, in Canada, the risk of acute myocardial infarction, stroke or heart failure is reported to be lower in South Asian patients with diabetes compared to White Caucasians [22].

The INTERHEART study reveals that approaches to CVD prevention can be based on similar principles worldwide [2]. In that study, diabetes contributed equally to the population attributable risk in people living in Western Europe and those living in the Middle East. A reason for the contradictory results, as compared with our study, could be that we are studying a population that has migrated. The distribution of CVD-associated risk factors may change during migration, as suggested by studies showing that migration per se may increase the prevalence of CVD-associated risk factors [6,7]. Moreover, our study presents a higher diabetes prevalence in Iraqi immigrants to Sweden compared to Iraqis living in Iraq (8.4 vs. 7.4%) [23], with diabetes developing after migration in most cases [24]. Consequently, the risk factor pattern for CVD may be altered after migration, and may change the impact of different risk factors on CVD risk. We conclude that longitudinal cohort studies are warranted to further study the impact of diabetes and other risk factors on CVD risk in populations that have migrated.

### Prevalence of diabetes in relation to country of birth

Immigrants from the Middle East represent a group at high risk of diabetes [8]. The prevalence of T2D in immigrants from Iraq and in Swedish-born participants correspond well with earlier reports reporting diabetes prevalences of 7% in Middle Eastern immigrant populations and of 4.4% in the general Swedish population [25].

Immigrants from the Middle East diagnosed with diabetes are also reported to have worse metabolic control compared to non-immigrants with diabetes [26]. Inadequate metabolic control in Iraqis with diabetes could theoretically contribute to faster progression to macrovascular complications and contribute to a higher prevalence of CVD, consistent with the observations in this study.

Iraq-born individuals account for a large proportion of the Swedish population [9]. We found indications of an increasing public health problem, with almost 40% of the Iraqi participants at moderate to high risk of developing diabetes within the next decade. The high diabetes risk, as estimated by the FINDRISC scores, is a consequence of a high proportion of Iraqi immigrants having clustering of diabetes risk factors such as higher weight, physical inactivity and family history of diabetes. Our data corresponds well with earlier reports of accumulation of obesity and physical inactivity in immigrants to Sweden from the Middle East [6].

### Hypertension and other CVD risk factors

Although risk factors for CVD such as T2D, abdominal obesity, physical inactivity, disturbed lipid metabolism (as estimated by an elevated p-LDL/p-HDL ratio) and psychosocial risk factors were more prevalent amongst Iraqi immigrants, blood pressure values were paradoxically lower than in Swedes. Our data are consistent with earlier studies reporting lower blood pressure values amongst immigrants from the Middle East compared to native Norwegians as well as compared to native Swedes [27,28]. In immigrants from the Middle East we also observed that CVD was more strongly associated with T2D than with history of hypertension which may indicate that diabetes may be a stronger risk factor for CVD than hypertension in this group. In Swedes, by contrast, participants with a history of hypertension had the highest OR for CVD.

Others have shown that education level increases the risk of CVD in non-immigrants [29] and that patients with diabetes living in deprived areas have a higher risk of CVD compared to those living in non-deprived areas [30]. Whereas the Swedish group gave results similar to those reported previously, in Iraqi participants we observed the opposite trend, with lower odds of CVD among those with education not more than high school exam (data not shown). We also assessed education level in the regression analysis, but in this survey it did not influence the outcome (data not shown).

### Strengths and limitations

A strength of this study is the careful matching of the Iraqi and Swedish cohorts living in the same socioeconomic neighborhood. Another strength is that we only considered participants reporting a history of diabetes as having diabetes only if they were currently taking diabetes medication and/or had fasting glucose levels within the limits of diabetes as having diabetes. One might argue that the lower proportion of Iraqi women compared to Swedish women (41.3 vs. 47.4%) is a limitation, but we do not think this biased the outcomes of our data analysis since gender was not an independent risk factor for CVD. Iraqis also had a higher participation rate as compared to Swedes and we consequently consider our main findings to be reliable.

We relied on self-reported data for CVD, since we did not have access to medical records from Iraq. Other limitations are that we do not have data on CVD prevalence from the background population not participating in the study. In the present study p-LDL/p-HDL ratio was used as a proxy for ApoB/ApoA, which was adjusted for in the INTERHEART study [2], and we did not include questions on locus of control [31], which was adjusted for in the INTERHEART study [2]. In this survey we failed to confirm that T2D was associated with CVD in participants born in Sweden. We conclude that this was a consequence of the lower power in this group. And although the prevalence of self-reported hypertension was lower in Iraqis versus Swedes (11 vs. 14%) the study may be underpowered to detect significant differences between the groups in the prevalence of hypertension.

Regarding the representativeness of the study sample, data on self-reported CVD amongst non-participants was not collected which is a limitation.

Another weakness is the inability to infer causality and the lack of information on disease onset for CVD, meaning that CVD onset may have preceded diabetes onset in some cases. However, we consider our conclusions to be reliable since T2D is a strong risk factor for CVD [20,32] while CVD is not a risk factor for T2D.

## Conclusions

In this survey, T2D contributed with higher odds of CVD in Iraqi immigrants compared to native Swedes. Our data collectively suggest that CVD prevention in immigrants from the Middle East in particular would benefit from the prevention of T2D. However, longitudinal studies are needed to identify mechanisms contributing to CVD risk in immigrants from the Middle East.

## Abbreviations

CVD: Cardiovascular disease; BMI: Body mass index; BP: Blood pressure; CI: Confidence interval; HDL: High-density lipoprotein; LDL: Low-density lipoprotein; OR: Odds ratio; PA: Physical activity; SD OR: Standardized odds ratio; T2D: Type 2 diabetes; TG: Triglycerides.

## Competing interests

The authors declare that they have no competing interests.

## Authors’ contributions

LB designed the study, wrote the research protocol, obtained, analyzed and interpreted the data, and wrote the manuscript. CDA assisted with interpreting the data and writing the manuscript. UL contributed to designing the study, interpreting the data and writing the manuscript. All authors have revised/edited the article critically and have approved the final version of the manuscript.

## Pre-publication history

The pre-publication history for this paper can be accessed here:

http://www.biomedcentral.com/1471-2458/13/1133/prepub
